# Increased Semantic Memorization in Children with ADHD during a Paradigm of Motor Priming: Exploratory Findings

**DOI:** 10.3390/children11070787

**Published:** 2024-06-28

**Authors:** Ana Moscoso, Clarisse Louisin, Simona Caldani, Mickael Worms Ehrminger, Mylene Fefeu, Eric Acquaviva, Richard Delorme, Maria Pia Bucci

**Affiliations:** 1 Child and Adolescent Psychiatry Department, Robert Debré Hospital, APHP & Université Paris Cité, Boulevard Sérurier, 75935 Paris, France; ana.moscoso@aphp.fr (A.M.);; 2 ICAR UMR 5191, CNRS, ENS de Lyon, Université Lyon 2, 69342 Lyon, France; 3 Centre for the Functional Exploration of Balance in Children (EFEE), Robert Debré Hospital, 75019 Paris, France; 4 Triptyque Scientific Consulting, Paris & Study Group REVER, 75010 Paris, France; 5 Human Genetics and Cognitive Functions, Institut Pasteur, 75015 Paris, France

**Keywords:** ADHD, children, learning, movement, verbal working memory

## Abstract

**Highlights:**

**What are the main findings?**

- Our paradigm suggested that listening to a story standing up while copying the experimenter’s movements that mimicked the actions told in the story significantly improved the semantic short-term memorization of participants.
- Movement priming may be a useful tool to improve auditory short-term memory in children with ADHD in challenging retention conditions.

**What is the repercussion of the main findings?**

- Our results could have repercussions for the management of children with ADHD at school, particularly in terms of the educational framework imposed during school time. More freedom of movement could facilitate learning for children with ADHD in the classroom.

**Abstract:**

Aim: The aim was to evaluate the effect of body actions on learning process, particularly semantic memory capabilities in drug-naïve children with attention deficit hyperactivity disorder (ADHD). Method: Thirty children had to listen to a story which was repeated three times in a row and then a fourth time five minutes later. After each listen, the child was asked what she/he remembered from the story. The whole sample was split randomly into three subgroups of equal IQ (mean 102.2 ± 12.7), age (mean age 8 ± 0.6 years), sex (ratio female to male 1:5) and severity of ADHD symptoms (34.2 ± 7.4); a G1 “Freeze” subgroup, which implied listening to the story while sitting on a chair without moving; a G2 “Minimal” subgroup, which implied listening to the story while sitting on a chair but free movement was allowed; a G3 “Prescribed movement” subgroup, which implied listening to the story standing up, while copying the experimenter movements that mimicked the actions told in the story. Results: Although our sample was limited in size, interestingly, children in the G3 subgroup showed the highest short-term semantic memory retention compared to G1. In all subgroups, repetition allowed an increase in performance. Conclusions: Our exploratory findings stress the positive role of movement in children with ADHD to increase semantic memorization. Hyperactivity may counteract the deficit of memorization related to attention impairment in children with ADHD. Our results may encourage parents or teachers to allow children with ADHD to move around during short-term memory-retention tasks.

## 1. Introduction

Attention deficit hyperactivity disorder (ADHD) affects around 5% of school-age children and is characterized by inattention associated or not with hyperactivity and impulsivity, which entails functional impairing in several settings of the individual’s life [1]. Presentation through the life span changes slightly, with younger children presenting more hyperactive symptoms than adults [2]. Research on academic challenges posed by ADHD indicates that individuals with this condition are more prone to experience higher rates of school retention, failed grades, school dropout and expulsion, special education referrals and problematic relationships with both peers and teachers [3,4].

Regarding explanatory factors, several studies reported associations between academic failure and vulnerabilities in executive and language processes such as information storage and working memory, phonological processing skills and awareness, syntax, fluency and spelling, leading to impairments in reading and/or writing [5,6].

For many years, hyperactivity by itself was also considered responsible for learning impairments, as calmness was deemed crucial for optimal attention performance. But this view has been challenged by a few authors who advocate that hyperactivity plays a functional role and serves the purpose of compensating for working memory relative hypoactivation while engaging in an academic cognitive task [7,8]. Specifically, the model postulates that challenges to underlying working memory components engender increased movement in children as a process that augments arousal, necessary for task performance [7]. The experimental assessment of this hypothesis was tested in children with ADHD during visual and verbal working memory tasks of increased difficulty. Although children with ADHD showed that better cognitive performances were associated with higher gross motor activity, this was not the case in their matched controls [9,10].

The crucial role of body movements in cognitive and learning processes has been documented in the literature [11,12]. Simulated actions can both prime and facilitate speech and cognition [11]. Movements can also serve as a memory enhancer, as demonstrated by studies conducted in both adults and children. In these studies, participants were asked to memorize a list of words while performing coherent prescribed hand gestures (visually related to the word itself). They obtained better scores than those whose gesturing was not solicited [11,13].

Not only do bodily functions influence cognition, but also, engaging in physical exercise potentially improves cognition. A growing number of studies have stated the virtues on cognitive functions of physical exercise, for everyone in general and individuals with ADHD in particular [14]. Sport seems to be beneficial in stimulating executive functions such as inhibition; flexibility; attention and working memory [2]; and emotional disturbances such as anxiety, depression or aggressivity [15]. However, results need to be read cautiously as exercise protocols often lack standardization and can be difficult to replicate.

These findings challenge the traditional learning principles implemented in schools, which emphasize the advantages of remaining stationary during the learning process such as sitting quietly on a school chair. Except for sport lessons, moving in class is often seen as ill-mannered and a source of complaints. If movement is demonstrated to play a significant role in cognition and has specific benefits, particularly in children with ADHD, it should be considered for inclusion into the classroom setting.

Our experiment builds on the existing literature and aims to test how different movement strategies change the semantic memorization abilities of children with ADHD. Given previous findings, our hypothesis is that movement will have a positive impact on these tasks.

## 2. Methods

### 2.1. Subjects

Thirty children with ADHD (age at inclusion: 8 ± 0.4 years; ratio female to male 1:5) were enrolled in the study at the Child and Adolescent Psychiatry Department at the University Robert Debré Hospital, APHP, France. They were diagnosed by experienced clinicians through clinical assessment following DSM-5 criteria [1]. The severity and diversity of symptoms were assessed with the ADHD-Rating Scale [16], and cognitive skills were explored using the Weschler scales adapted to age. To be included in the study, children had to be diagnosed with ADHD, drug-naïve, aged between 6 and 10 years, with all sub-scores of the intellectual quotient within the normal range (80–120) and had to be a native French speaker. Children with a comorbidity of dysphasia, severe coordination disorder and/or other major psychiatric mental disorders or neurodevelopmental disorders (including bipolar disorder, major depression and autism) were excluded from the study.

### 2.2. Ethics

The investigation followed the principles of the Declaration of Helsinki and was approved by our Institutional Human Experimentation Committee (Comité de Protection des Personnes CPP Île-de-France I (N° IDRCB: 2021-A00489-32). Written informed consent was obtained from the children’s parents after the experimental procedure was explained to them. Additionally, consent was obtained from the children, in a manner appropriate for their age and comprehension level.

### 2.3. Material

For the purpose of this study, we used an adaptation of the “market game” test [17], adapted to the age of the enrolled subjects, which assesses attention and short-term memory abilities. This game consists of a small narrative that enlists several fruits and vegetables bought by the main character at a market. Children are asked to recall which fruits and vegetables these were 5 min after listening to the story. The interval between listing to the story and recalling is spent playing games that make little recruitment of executive resources. In the present study, the story was changed to avoid test–retest effects and to elicit both working memory and short-term memory abilities. Here, the short story consisted of a “visit to the park” in which the main character performs several activities or actions that are coherent with the setting (Table 1). The actions contained in the story were then solicited by the investigator three times immediately and once 5 min later. While immediate recall elicits mainly working memory, 5-min-later recall requires short-term memory [18]. The story was repeated three times in a row to test for learning consolidation.

### 2.4. Experimental Procedure

Participants were divided into three subgroups of equal IQ, sex, age and ADHD-RS total score (Table 2). The G1 ‘*freeze*’ subgroup included 10 children displaying no movement i.e., they were asked to stay seated on a chair without moving behind a desk in front of the experimenter. The G2 ’*Minimal*’ subgroup included 10 children who were allowed to move on the chair behind a desk while listening to the story. The G3 ‘*Prescribed movement*’ sub-group included 10 children standing up who had to reproduce the gestures performed by the experimenter. These movements, which mimicked the actions described in the story, were meant to serve as a motor primer, accompanying the narrative.

The experimenter was blind to the hypothesis of the study. The experience was run in a quiet and isolated room. Each group listened to the story three times. After each retelling, the participant was immediately asked to repeat the words/verbs in the story that they remembered. Following the three listening sessions, a 5 min break took place. During this time, the child would engage in small talk with the experimenter, discussing favorite foods, activities or friends at school. After 5 min, the experimenter would solicit once more the recall of actions contained in the story without telling the story.

### 2.5. Data Analysis

The experimenter counted the words/verbs remembered by the participant after each of the 3 listening sessions and once 5 min after the third session. If the child remembered all the words/verbs, the total score was 17.

### 2.6. Statistical Analysis

Given the small sample size and the study design, descriptive statistics are reported as the median [1st quartile; 3rd quartile]. We analyzed the data with Kruskal–Wallis tests for independent groups [19]. The statistical analysis procedure was applied as follows: (i) within each group (G1, G2, G3) for descriptive and illustrative purposes, (a) the median score for each of the four data collection (T1, T2, T3, T4) points was computed, as well as the interquartile range, (b) and a learning curve was drawn; (ii) between groups, (a) Kruskal–Wallis analyses were performed on the scores at each timepoint to check for the existence of global intergroup differences. When such global intergroup differences were found, post hoc Dunn–Bonferroni analyses were performed to compare the groups pairwise.

A first significance threshold was set at 0.05 for the initial Kruskal–Wallis analyses and was then corrected to adjust for multiple comparisons (0.05/3 = 0.0167).

Spearman correlations between IQ and semantic performance were additionally tested to verify for potential confounding factors.

## 3. Results

### 3.1. At Timepoint 1

**Figure 1:**
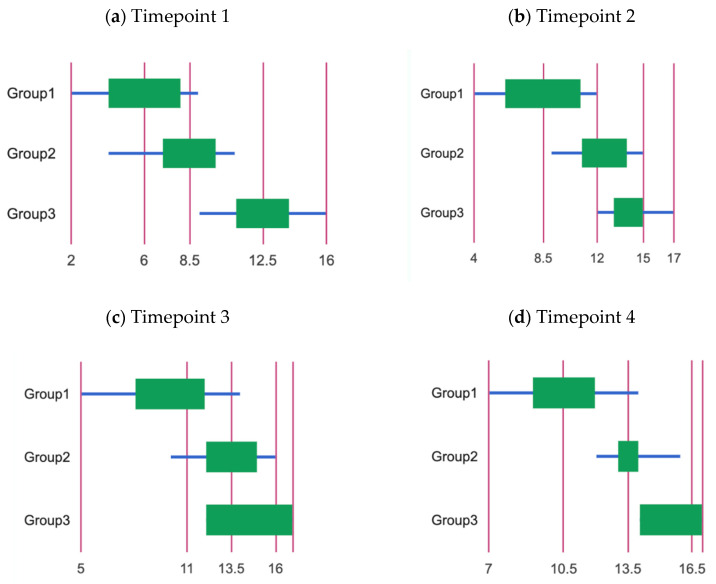
Semantic memory performance in the three studied groups (G1, G2, G3) at the four timepoints. (**a**) Timepoint 1, (**b**) Timepoint 2, (**c**) Timepoint 3, (**d**) Timepoint 4.

The Kruskal–Wallis H test indicated that there was a significant difference in the dependent variable between the different groups, χ^2^(2) = 19.11, *p* < 0.001, with a mean rank score of 7.6 for Group 1, 14.3 for Group 2 and 24.6 for Group 3. The post hoc Dunn’s test indicated that the mean ranks of the following pairs were significantly different: Group 1 vs. Group 3 (*p* < 0.001) and Group 2 vs. Group 3 (*p* < 0.01) (Figure 1a). The general effect size across the groups was high (eta-square = 0.63).

### 3.2. At Timepoint 2

The Kruskal–Wallis H test indicated that there was a significant difference in the dependent variable between the different groups, χ^2^(2) = 18.11, *p* < 0.001, with a mean rank score of 6.9 for Group 1, 16.1 for Group 2 and 23.5 for Group 3. The post hoc Dunn’s test indicated that the mean rank of the following pair was significantly different: Group 1 vs. Group 3 (*p* < 0.001) (Figure 1b). The general effect size across the groups was high (eta-square = 0.6).

### 3.3. At Timepoint 3

The Kruskal–Wallis H test indicated that there was a significant difference in the dependent variable between the different groups, χ^2^(2) = 12.31, *p* = 0.002, with a mean rank score of 8.1 for Group 1, 16.8 for Group 2 and 21.6 for Group 3. The post hoc Dunn’s test indicated that the mean rank of the following pair was significantly different: Group 1 vs. Group 3 (*p* < 0.001) (Figure 1c). The general effect size across the groups was moderate (eta-square = 0.38).

### 3.4. At Timepoint 4

The Kruskal–Wallis H test indicated that there was a significant difference in the dependent variable between the different groups, χ^2^(2) = 18.61, *p* < 0.001, with a mean rank score of 7.2 for Group 1, 15.35 for Group 2 and 23.95 for Group 3. The post hoc Dunn’s test indicated that the mean rank of the following pair was significantly different: Group 1 vs. Group 3 (*p* < 0.001) (Figure 1d). The general effect size across the groups was high (eta-square = 0.62).

### 3.5. Learning Curves

**Figure 2:**
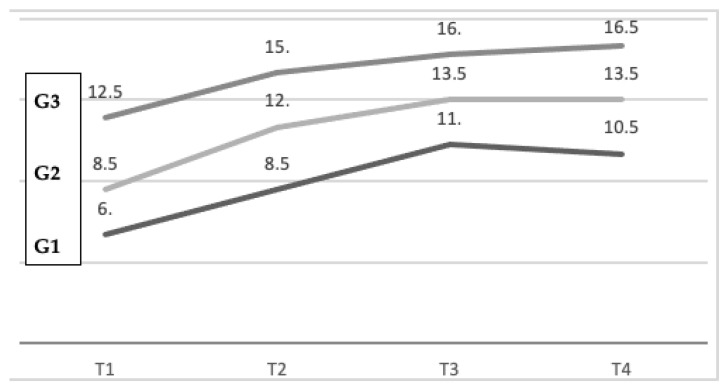
Learning curves of the three studied conditions. Median evolution at the four timepoints.

The learning curves of the three groups (Figure 2) showed an improvement in semantic performances in all three groups, from T1 to T3. Progression from T3 to T4, when short-term memory was tested, was marked by loss (G1), no evolution (G2) or improvement (G3). Given these results, we can suppose Group 3 (prescribed movement) performed significantly better than Group 1 (freeze) at all timepoints. Group 3 performed better than Group 2 (minimal movement) after the first listening session. No significant difference could be evidenced at all timepoints between the latter groups, which could be explained by the low statistical power of the study.

### 3.6. Confounding Factors Analysis

Spearman correlation coefficients were calculated between IQ and tested performances for the whole sample (N = 30) to verify for potential confounders (Table 3). The results showed no correlations between individual IQ and performances, except for the working memory subscales at T1 (*p* = 0.010) and T2 (*p* = 0.038).

## 4. Discussion

Our experiment is keen to deliver two preliminary findings.

The inclusion of drug-naïve children ensured that the findings were not confounded by medication effects, offering greater insight into the natural interactions between movement and memory in children with ADHD.

*First, there was a significant group effect in semantic memory recall*, with group G3 (augmented movement group) systematically getting better scores compared to G1 (freeze group); better performances were observed in G3 compared to G2 (minimal spontaneous movement group) only at the first timepoint. Our results build on findings from other authors, such as [9], that observed how higher rates of gross motor activity positively predicted phonological working memory performances for children with ADHD in experimental conditions, or [10], who obtained similar results in another working memory task. They suggested that being able to move may lead to optimal arousal and performance improvement in children with ADHD. Moreover, in G1, there was an imposition of staying still. Note that this mode of learning is often observed at school. The results are also aligned with other authors that showed how, because being instructed not to gesture is itself a cognitive load, subjects remember fewer items [20]. The act of gesturing lightens learners’ cognitive load, allowing subjects to work harder on the task and perhaps change the representation of the task [10,12].

The motor primer proposed in G3, where subjects copied the movements performed by the investigator, served the purpose of enhancing short-term memory. Our results suggest improvement, and the findings are aligned with [11,13], where participants were asked to memorize a list of words while performing coherent prescribed gestures (visually related to the word itself), obtaining better scores than those to whom this was not prescribed.

Additionally, movement reproduction implied looking at the researcher to perform the same gestures, which implied ocular fixation, also facilitated by attention and other cognitive competencies [21], and this might be beneficial for ADHD individuals where attention is challenging. In this task, augmented learning was fueled through kinesthetic short-term memory competencies (i.e., the ability to remember and replicate movements or physical actions), with a possible synergic effect. Kinesthetic memory is also sensible to rehearsal and has been used successfully in learning processes [22]. At a structural level, this type of learning displays—partly—the activation of mirror neurons, implied in imitative responses of motor actions [23].

*Second, repetition allowed for a progressively significant increase in semantic working memory performance in all the three ADHD groups*, from T1 to T3. Our results suggest that children with ADHD are sensible to repetition and recall increasingly larger amounts of data if repeated. Progression from T3 to T4, when short-term memory was tested, was marked by loss (G1), no evolution (G2) or improvement (G3). If, in a particular experimental procedure, no loss of short-term memory (as measured at timepoint 4) is observed, one can attribute that response pattern to rehearsal [18]. This was the case with G3, which could have been more sensitive to rehearsal than the other groups. However, given the small sample size and the statistical power, this result must be confirmed by larger studies.

Given working memory deficits are present in a substantial proportion of children with ADHD, and converging evidence links these deficits with ADHD-related functional learning impairments [24], this result could be somewhat unexpected. At the same time, working memory is not a unitary construct, and is more likely a system of several components [25]. Some studies indicate that the ability to display instant memory recall (as defined by this study protocol in T1, T2 and T3) might be vehiculated through the episodic buffer, likely intact in these children [25,26]. Our results lack, however, the comparison with a control group tested under the same conditions.

Confounding factor analysis indicates correlations between individual working memory abilities and the first two listening sessions (T1 and T2) but not the sessions after (T3 and T4). No other correlation was found between other IQ subscales, total IQ and performances. Even if individual working memory abilities modulate performances in the first stages of the experiment (where verbal working memory is redundantly tested), any potential effects disappear after, suggesting movement condition effects.

## 5. Study Limitations

The inclusion of drug-naïve children ensured that the findings were not confounded by medication effects, offering a clearer view of the natural interactions between movement and memory in children with ADHD. However, our results require validation, as the experiment was run without a control group and limited to 30 participants. Given the low power (small sample size and multiple comparisons), caution is warranted to draw definitive conclusions. Our goal is to overcome these limitations in future studies by including comparisons with matched controls of children without ADHD and by increasing the number of tested subjects. Future research will also aim to explore the existence of age-related optimal movement levels and task optimization.

## 6. Conclusions

Our preliminary results indicate that movement could play a beneficial role in learning and memorization in children with ADHD. They underline the benefits of gesturing and movement priming when performing a solicited verbal working memory and short-term memory task. These findings are integrated in the context of the intrapersonal cognitive and facilitatory effects of gestures and underline the correlation between language, action and cognition. Again, they challenge the traditional learning principles implemented in schools, which emphasize the advantages of remaining quiet during the learning process (for instance, sitting quietly in a chair). If movement is demonstrated to play a significant role in cognition and has clear benefits, particularly in children with ADHD, we should consider its integration in the classroom setting.

## Figures and Tables

**Table 1:** Test description: a visit to the park. The investigator read the story to the child (30′ duration). The name of the child in the test was changed and became the name of the subject to stimulate better retention.

‘Yesterday the child went to the park, and he did…. Cycling, he climbed on a tree, jumped on the sandbox, lay down in the grass, then fell asleep looking at the clouds. He woke up to go slide down the yellow slide and back home running to get his snack’.

**Table 2:** Clinical characteristics of the three subgroups of children with ADHD (n = 30). Continuous variables at mean ± standard deviation.

	G1 Freeze	G2 Minimal	G3 Prescribed Movement
	(n = 10)	(n = 10)	(n = 10)
**Age at inclusion** (years) (SD)	8.1 ± 0.4	8.5 ± 0.4	8.2 ± 0.6
**ADHD-Rating scale mean (SD)**			
Total score	34.1 ± 2.5	35.2 ± 2.5	33.0 ± 2.0
Inattention sub-score	16.9 ± 1.6	18.5 ± 1.1	16.7 ± 1.2
Hyperactivity/impulsivity sub-score	19.4 ± 1.8	20.1 ± 2.3	17.1 ± 1.9
**Wechsler scale**			
Mean Total IQ (SD)	103.6 ± 8.5	100 ± 15.5	105.7 ± 13.8
Median Total IQ	101.5	100	108

**Table 3:** Non-parametric correlations between IQ and performances at four timepoints, with all subjects (N = 30) confounded. * highlights statistically significant associations (*p* < 0.05).

		T1	T2	T3	T4
Verbal subscale	Spearman’s rho	−0.072	−0.115	−0.027	−0.128
	*p*-value	0.709	0.554	0.891	0.508
Visuo-spatial subscale	Spearman’s rho	0.117	0.079	0.145	0.063
	*p*-value	0.545	0.682	0.454	0.745
Perceptual reasoning subscale	Spearman’s rho	0.279	0.114	0.107	0.114
	*p*-value	0.143	0.554	0.579	0.555
Working memory subscale	Spearman’s rho	0.424	0.424	0.335	0.353
	*p*-value	0.022 *	0.022 *	0.076	0.060
Processing speed subscale	Spearman’s rho	0.304	0.348	0.251	0.339
	*p*-value	0.109	0.065	0.190	0.072
Total IQ	Spearman’s rho	0.217	0.156	0.126	0.123
	*p*-value	0.258	0.420	0.513	0.526

## Data Availability

Data is available from authors upon reasonable request.
